# Combinatorial blockade for cancer immunotherapy: targeting emerging immune checkpoint receptors

**DOI:** 10.3389/fimmu.2023.1264327

**Published:** 2023-10-19

**Authors:** Dia Roy, Cassandra Gilmour, Sachin Patnaik, Li Lily Wang

**Affiliations:** ^1^ Department of Translational Hematology and Oncology Research, Cleveland Clinic Foundation, Cleveland, OH, United States; ^2^ Department of Molecular Medicine, Case Western Reserve University School of Medicine, Cleveland, OH, United States

**Keywords:** immune checkpoint inhibitors, combinatorial immunotherapies, PD-1, CTLA-4, VISTA, TIGIT, TIM3, LAG3

## Abstract

The differentiation, survival, and effector function of tumor-specific CD8^+^ cytotoxic T cells lie at the center of antitumor immunity. Due to the lack of proper costimulation and the abundant immunosuppressive mechanisms, tumor-specific T cells show a lack of persistence and exhausted and dysfunctional phenotypes. Multiple coinhibitory receptors, such as PD-1, CTLA-4, VISTA, TIGIT, TIM-3, and LAG-3, contribute to dysfunctional CTLs and failed antitumor immunity. These coinhibitory receptors are collectively called immune checkpoint receptors (ICRs). Immune checkpoint inhibitors (ICIs) targeting these ICRs have become the cornerstone for cancer immunotherapy as they have established new clinical paradigms for an expanding range of previously untreatable cancers. Given the nonredundant yet convergent molecular pathways mediated by various ICRs, combinatorial immunotherapies are being tested to bring synergistic benefits to patients. In this review, we summarize the mechanisms of several emerging ICRs, including VISTA, TIGIT, TIM-3, and LAG-3, and the preclinical and clinical data supporting combinatorial strategies to improve existing ICI therapies.

## Introduction

The cancer-immunity cycle refers to the process wherein tumor antigen-reactive T cells undergo successful priming and differentiate into cytotoxic killer T cells that infiltrate tumor tissues and eliminate cancer cells (1). The differentiation, expansion, survival, and effector function of these tumor-specific cytotoxic T cells (CTLs) is regulated by the collective signaling effects of the T-cell receptor, costimulatory/coinhibitory receptors, and cytokine receptors, which culminate in transcriptional and epigenetic programs to guide T-cell fate. Unlike in acute viral infections where effector CTLs and memory T-cell responses develop properly, tumor-specific CTLs exhibit dysfunctional states in response to chronic stimulation and a myriad of immunosuppressive factors in the tumor microenvironment (TME). These T cells progressively lose proliferative capacity, memory potential, and effector functions, and enter an “exhausted” state. Exhausted T cells upregulate the expression of multiple ICRs, including PD-1, CTLA-4, VISTA, TIGIT, TIM-3, and LAG-3, which sustain dysfunctional antitumor T-cell responses (2, 3).

Immune checkpoint inhibitors (ICIs) are antibodies or small molecules that bind and block the function of ICRs, thereby reducing tumor-induced T-cell exhaustion and restoring anticancer immunity. Ipilimumab, the monoclonal antibody (mAb) blocking cytotoxic T lymphocyte antigen 4 (CTLA-4), was the first ICI therapy approved by the Food Drug Administration (FDA) in 2011. Currently, several mAbs targeting CTLA-4, PD-1, and PD-L1 have been approved for clinical applications. However, despite revolutionizing the field of oncology, the major challenge of existing ICI therapies is the overall low response rate. Understanding the unique molecular and cellular mechanisms of each ICR may support the development of novel combinatorial therapies that optimally restore antitumor immunity.

**Figure 1:**
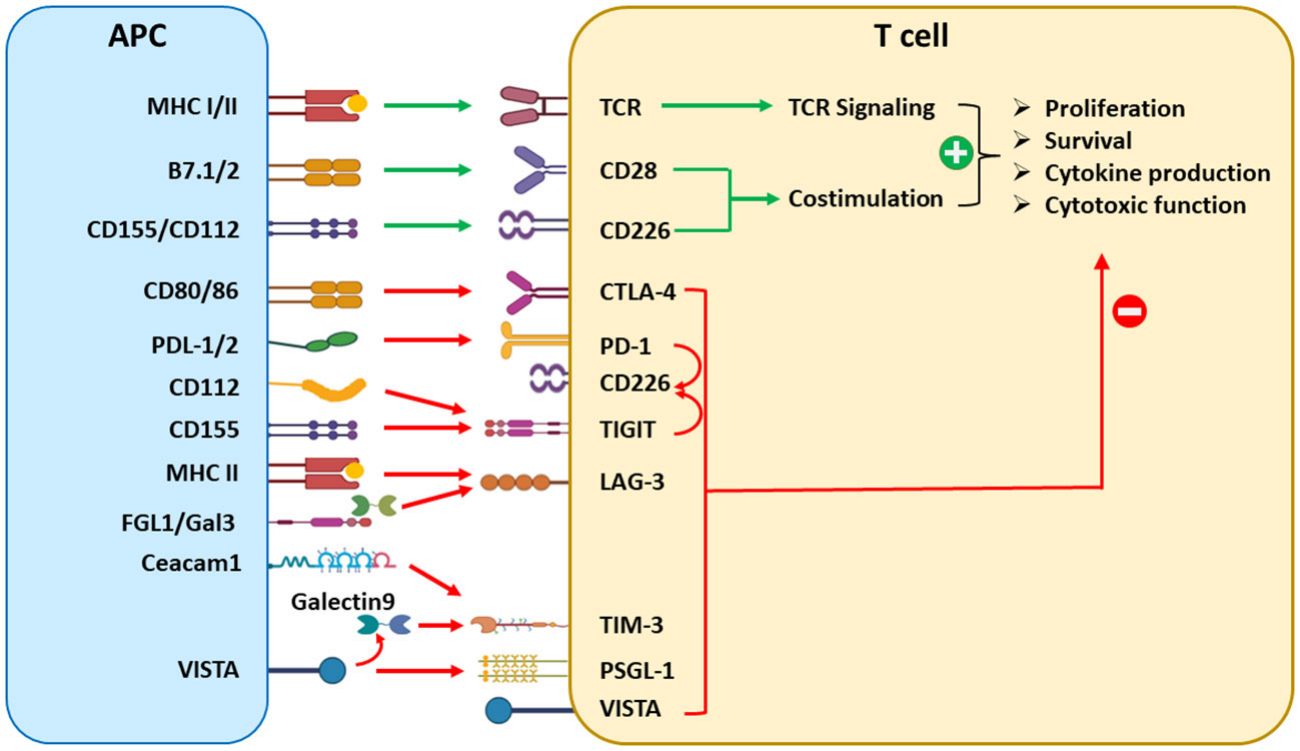
Overview of coinhibitory ICRs and their effects in conventional T cells. T-cell activation requires TCR recognition of cognate antigens presented on APCs and costimulation provided by B7/CD28 or CD115/CD226 interactions. On the other hand, many coinhibitory ligand/receptor pathways are activated to dampen T-cell responses. The B7/CTLA-4 and PD-L1/2/PD-1 pathways are the cornerstones of the immune checkpoint paradigm. Emerging inhibitory ICRs, including TIGIT, LAG-3, TIM-3, and VISTA, each recognized by multiple ligands, play nonredundant yet convergent roles as the “brakes” of T-cell responses.

**Figure 2:**
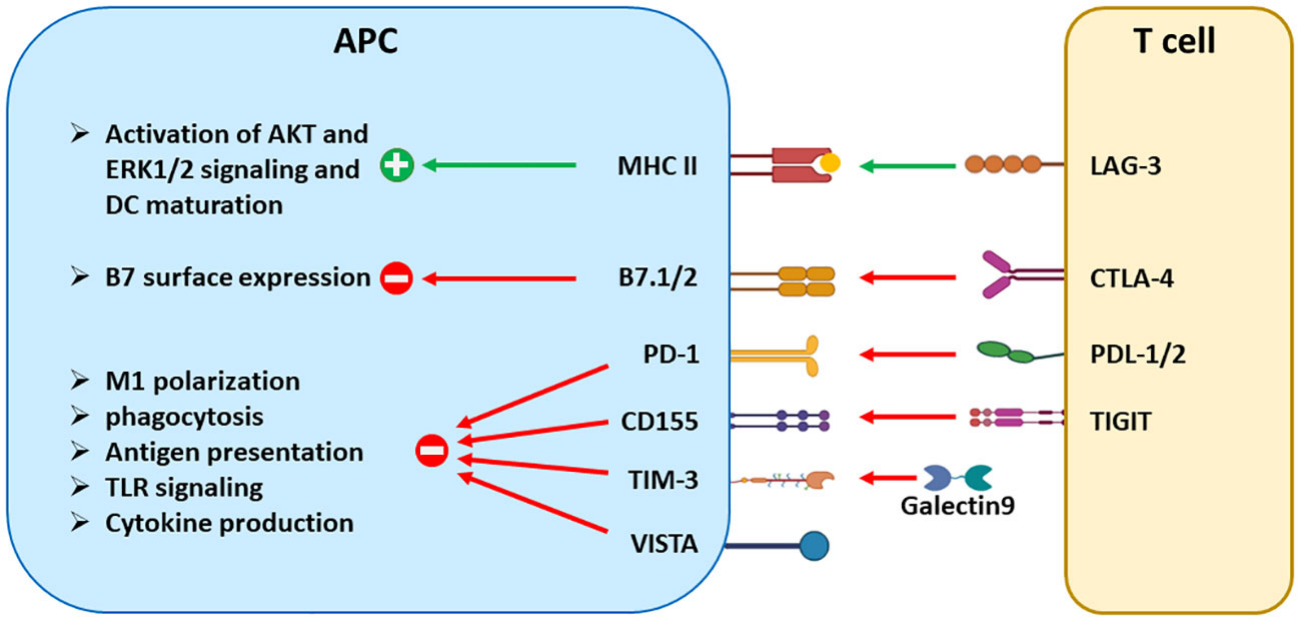
The signaling effects of ICRs in antigen-presenting cells. Aside from suppressing T-cell activation, many ICRs regulate the maturation, antigen presentation, cytokine production, and other effector functions of DCs and tumor-associated macrophages. CTLA-4 reduces the surface expression of B7 molecules through trans-endocytosis. LAG-3 and TIGIT trigger signaling in a reverse direction by engaging their respective binding partners MHCII and CD155. On the other hand, PD-1, TIM-3, and VISTA are expressed in APCs and transmit inhibitory signals to inhibit the effector functions of APCs, including phagocytosis, antigen presentation, and cytokine production. Both PD-1 and VISTA are also expressed in tumor-driven MDSCs and contribute to the differentiation and suppressive function of MDSCs.

This review summarizes updated literature regarding the established and emerging ICRs: PD-1, CTLA-4, VISTA, TIGIT, TIM-3, and LAG-3. Due to the scope limitation, we omit discussions of additional emerging ICRs such as B7-H3, B7-H4, HHLA2, and butyrophilin-like 2 (BTNL2), which have been reviewed elsewhere (4). Herein, we provide an overview of each ICR’s structure, expression, signaling mechanisms, and current preclinical and clinical data. We also elaborate on the concept that multiple ICRs operate concurrently to impair the expansion, survival, and effector functions of tumor-reactive cytotoxic T cells (**Figure 1**), as well as control the maturation and function of dendritic cells (DCs), macrophages, and myeloid-derived suppressor cells (MDSCs) (**Figure 2**). Given the frequent coexpression and functional crosstalk of these ICRs, we affirm the concept that combinatorial targeting of ICRs may achieve synergistic therapeutic outcomes compared to monotherapies.

## Programmed death -1

### PD-1 structure and expression

Programmed death -1 (PD-1, CD279) belongs to the B7/CD28 family of receptors, which are type-I transmembrane proteins consisting of an immunoglobulin variable (IgV) domain, a transmembrane domain, and a cytoplasmic tail with signaling capacities. PD-1 engages the ligands PD-L1 and PD-L2 and acts as a coinhibitory receptor that regulates both the adaptive and innate arms of the immune system (4, 5).

PD-1 expression is detected in activated T cells, Foxp3^+^ regulatory T cells (Tregs), natural killer (NK) cells, innate lymphoid cells (ILC2s), B lymphocytes, macrophages, DCs, and monocytes. In T cells, PD-1 gene expression is induced by TCR signaling and positively regulated by multiple transcription factors including AP-1, NFATc1, FoxO1, NF-_K_B, Notch, STAT, and IRF9 (5). In cancers and chronic viral infections, PD-1 expression in exhausted T cells is significantly higher than in T cells from healthy hosts (3). The expression of PD-1 and its ligand PD-L1 on immune cells and cancer cells may serve as an indicator of disease progression and poor prognosis in a wide range of cancers (6).

### Molecular mechanisms of PD-1

The intracellular domain of PD-1 contains an immunoreceptor tyrosine-based inhibitory motif (ITIM) and an immunoreceptor tyrosine-based switch motif (ITSM) (5). In T cells, the engagement of PD-1 by its ligand PD-L1 results in the recruitment of the tyrosine-protein phosphatases SHP1 and SHP2, which downregulate the phosphoinositide 3-kinase (PI3K), mitogen-activated protein kinase (MAPK), and mammalian target of rapamycin (mTOR) pathways. CD28 can be directly dephosphorylated by SHP2 and is the major target of PD-1 inhibitory signaling (7). At the cellular level, the consequences of the PD-1 pathway are multifaceted, resulting in altered T-cell metabolism with impaired glycolysis and augmented fatty acid oxidation, reduced cell expansion and effector cytokine production, and impaired T-cell mobility (3, 4).

In addition to the canonical PD-L1/PD-1 interactions, PD-L1 binds to CD80, which is expressed on antigen-presenting cells (APCs) and activated T cells (8). Trans-interactions of PD-L1 on APCs and CD80 on T cells could transmit inhibitory signaling to T cells and impair antitumor immunity (8, 9). On the other hand, cis-interactions of PD-L1/CD80 on APCs reduced PD-L1/PD-1 interactions and CD80/CTLA4 interactions, without affecting interactions between CD80 on APCs and CD28 on T cells (10–12). Blocking cis-interaction of PD-L1/CD80 reduced CD80 expression on APCs and impaired antitumor immune responses (11). An anti-CD80 antibody blocking PD-L1/CD80 cis-interactions augmented PD-L1/PD-1 interactions and alleviated autoimmune disease (13).

In addition to T cells, PD-1 is expressed in tumor-associated macrophages and inhibits their phagocytic function, which in turn controls antitumor immune responses (14). Furthermore, PD-1 plays a role in regulating tumor-driven emergency myelopoiesis. PD-1 deletion in myeloid progenitors reduced the accumulation of GMPs and MDSCs, which may be the result of elevated ERK1/2 and mTORC1 signaling and metabolic reprogramming (15). In preclinical models and cancer patients, blocking interactions of PD-1 with PD-L1 augments the effector function of PD-1^+^ exhausted CTLs, and induces the expansion of TCF1^+^ progenitor-like exhausted T cells with self-renewal capacity (16). On the other hand, blocking PD-1 may trigger hyperproliferation and suppressive function of Tregs and contribute to hyperprogressive diseases (17).

### Targeting the PD-1/PD-L1 axis for cancer immunotherapy

Monoclonal antibodies specific for PD-1 (nivolumab, pembrolizumab), and PD-L1 (durvalumab, atezolizumab, and avelumab) have proven to be clinically effective and gained FDA approval across a wide range of cancers, such as skin cancer, lung cancer, Hodgkin lymphoma, renal cell carcinoma (RCC), head and neck cancer, bladder cancer, colorectal cancer, liver cancer, gastric cancer, triple negative breast cancer, and cervical cancer (18, 19). Additional antibodies blocking PD-1, such as cemiplimab, camrelizumab, sintilimab, toripalimab, tislelizumab, zimberelimab, prolgolimab, and dostarlimab, have been approved for cancer applications worldwide. A meta-analysis of randomized controlled trials has concluded that anti-PD-1/PD-L1 inhibitors are more advantageous for treating advanced and metastatic cancers than conventional therapies, with better overall survival and progression-free survival particularly in male patients with younger age, without central nervous system or liver metastasis, no EGFR mutations, and with higher PD-L1 expression (18).

While PD-L1/PD-1 inhibitors are approved for treating an expanding list of cancers, their use as monotherapies generated an overall low response rate, due to mechanisms of primary and acquired resistance (20, 21). To improve the response rate to ICIs, numerous combination strategies have been studied in preclinical and clinical trials, including combining PD-L1/PD-1 inhibitors with chemotherapeutics such as cyclophosphamide, radiotherapy, targeted therapy, agonistic costimulatory antibodies targeting CD134, CD137 or ICOS, innate immune stimulators such as STING agonists, epigenetic modulators, and cancer vaccines such as oncolytic viruses (19, 22, 23). On the other hand, these combinatorial regimens fail to address the roles of other non-overlapping ICRs that constitute one of the dominant resistance mechanisms to PD-1/PD-L1 inhibitors. In the rest of this review, we will summarize studies of emerging ICRs (i.e., VISTA, TIGIT, TIM-3, and LAG-3) and demonstrate the rationales for combinatorial therapies targeting non-redundant ICRs together with PD-1/PD-L1 inhibitors.

## CTLA-4

### CTLA-4 structure and expression

Cytotoxic T lymphocyte-associated protein 4 (CTLA-4, CD152), together with CD28, represents the B7 family of receptors. Similar to PD-1, CTLA-4 contains an extracellular IgV-domain, a transmembrane domain, and a cytoplasmic tail with motifs for intracellular signaling (24, 25). CTLA-4 is constitutively expressed on Foxp3+ regulatory T cells (Tregs) and is inducible upon activation in conventional T cells. In addition, CTLA-4 expression has been detected in natural killer cells, B cells, dendritic cells, and myeloid cells (26–31).

In T cells, CTLA-4 gene expression is induced by Foxp3 and NFAT (32). The stability of CTLA-4 mRNA is regulated post-transcriptionally, by microRNAs such as miR-145 and miR-155 (33, 34). In resting T cells, a majority of CTLA-4 resides intracellularly within endosomes and relocalizes to the cell surface upon TCR stimulation (27, 31, 35–37). CTLA-4 protein localization is dynamically regulated by clathrin-mediated endocytosis and endosomal recycling, which is dependent upon the tyrosine phosphorylation status of its cytoplasmic domain (38).

### Molecular mechanisms of CTLA-4

CTLA-4 inhibits the expansion, cytokine production, and differentiation of conventional T cells and contributes to the development and function of Foxp3^+^ Tregs. CTLA-4 exerts inhibitory effects by competing against CD28 due to its higher affinity for B7 molecules, as well as by recruiting phosphatases SHP2 and PP2A, which in turn downregulate signaling of TCR and CD28 (39–42). In addition to T-cell intrinsic mechanisms, CTLA-4 indirectly suppresses T-cell responses by modulating dendritic cells: CTLA-4 downregulates the surface expression of B7 molecules through trans-endocytosis (43) or induces the expression of indoleamine 2,3-dioxygenase (IDO), which in turn impairs T-cell proliferation (44). CTLA-4 also reverses the stop signal in activated T cells and reduces the contact time between T cells and APCs, leading to decreased cytokine production and T-cell proliferative responses (45).

The mechanisms of CTLA-4-mediated immunosuppression in cancers are distinct from PD-1 and potentially synergistic with PD-1 (46): although both receptors act on activated conventional T cells, PD-1 controls effector T-cell function at a later stage, mainly within peripheral tissue sites and the tumor microenvironment, while CTLA-4 intercepts T-cell priming in the lymph nodes and governs the function of Tregs (47, 48). CTLA4 is constitutively expressed in Foxp3^+^ Tregs and CTLA-4-specific antagonistic antibodies not only augment effector T-cell activation but also induce ADCC-mediated depletion of tumor-infiltrating Tregs (49–51). On the other hand, unlike PD-1 and PD-L1, CTLA-4 is not expressed in myeloid cells and does not directly regulate suppressive myeloid cells within the TME. These functional distinctions provide mechanistic rationales for developing combination therapies targeting both axes.

### Combinatorial blockade of PD-1 and CTLA-4

Studies have shown that while CTLA-4 and PD-1 blockade each boosts antitumor T-cell responses, dual blockade results in stronger therapeutic outcomes in preclinical models and human patients (52–54). ICI monotherapies induced the expansion of different tumor-infiltrating T cells (TILs), i.e., PD-1 blockade expanded exhausted-like CD8^+^ CTLs, whereas CTLA-4 blockade expanded both ICOS^+^ Th1-like CD4 effectors and exhausted CD8^+^ CTLs. In contrast, the combined blockade induced the expansion of terminally differentiated effector CD8+ CTLs that are not seen in monotherapies and further increased Th1-like CD4^+^ effector T cells (52, 53). Similar findings have been shown in human melanoma patients treated with ipilimumab and nivolumab therapy. In addition to melanoma, dual blockade of CTLA-4 and PD-1 was studied in a murine breast cancer model (53). While monotherapies showed modest effects, combination therapy led to complete tumor regression in a majority of mice. The synergistic efficacy was due to the anti-CTLA-4 antibody-induced expansion of the T-cell receptor (TCR) repertoire and augmented functionality of TILs, accompanied by intratumoral Treg depletion. Taken together, these studies have demonstrated the mechanisms of synergy with dual ICI therapy that may guide clinical applications.

Ipilimumab (Yervoy) was the first FDA-approved monoclonal antibody for cancer immunotherapy, owing to robust clinical responses for metastatic melanoma (55, 56). We summarize recent clinical trials that have advanced PD-1 and CTLA-4 combinatorial therapy; comprehensive overviews of other clinical trials involving ipilimumab can be found in other reviews (57, 58). As a monotherapy, the effect of ipilimumab is not as strong as that of the PD-1 antibody nivolumab (Opdivo) for resected stage III or IV melanoma and showed shorter survival and higher toxicity for patients than the PD-1 antibody pembrolizumab (Keytruda) (59, 60). However, when ipilimumab was given concurrently with PD-1 antibody, dual blockade therapy demonstrated significantly improved outcomes in clinical studies. The advantages of dual ICI therapy were first noted in a Phase I dose-escalation study using nivolumab and ipilimumab administered together, which led to better response rates and progression-free survival compared to previously reported results from either monotherapy (61). A subsequent phase III study highlighted better responses and survival with combinatorial therapy when used for metastatic melanoma patients with PD-L1 negative tumors compared to either nivolumab alone or ipilimumab alone, despite the higher occurrence of grade 3 or 4 treatment-related adverse events (62). Follow-up studies showed durable responses and sustained benefits for survival in these patients across multiple years (63–65). Treatment-naive patients with advanced melanoma also benefited from nivolumab-plus-ipilimumab treatment, once again producing higher objective-response rates and progression-free survival with acceptable safety profiles compared to ipilimumab alone (57).

Current research continues to advance PD-1 and CTLA-4 combinatorial immunotherapy in the treatment of other cancers. Beyond melanoma, FDA approval of anti-PD-1 and anti-CTLA-4 dual therapy has expanded to hepatocellular carcinoma (HCC), unresectable pleural mesothelioma, RCC, metastatic non-small cell lung cancer (NSCLC), and advanced or metastatic esophageal squamous cell carcinoma (66–68). Combinatorial ICI therapy in the neoadjuvant setting has also shown promise, with tolerance and strong pathological responses for late-stage melanoma, early-stage colon cancers, and late-stage urothelial cancer (69, 70). Dual blockade of CTLA-4 and PD-1 is currently being evaluated in numerous clinical trials for advanced solid tumors, such as head and neck squamous cell carcinoma (HNSCC) and glioblastomas (NCT04080804, NCT04606316). For testing combined treatment with pembrolizumab (anti-PD-L1), a randomized, double-blind phase III KEYNOTE-598 study (NCT03302234) showed that in patients with metastatic NSCLC, adding ipilimumab to pembrolizumab did not improve efficacy and exhibited greater toxicity than pembrolizumab monotherapy (71). Another phase I expansion trial (NCT02089685) evaluated the efficacy and safety of pembrolizumab combined with a reduced dose of ipilimumab in patients with advanced melanoma and RCC and showed manageable toxicity profile and robust antitumor activity (72).

## VISTA

### VISTA structure and expression

V-domain immunoglobulin suppressor of T-cell activation (VISTA, alias Gi24, Dies-1, PD-1H, DD1α) is homologous to B7 family receptors and acts as a negative regulator of antitumor immunity and autoimmunity (73–78). VISTA is a type I transmembrane protein containing a single IgV-like extracellular domain (ECD), a transmembrane segment, and a cytoplasmic tail that does not contain ITAM, ITIM, or ITSM motifs. Structural studies have revealed unique features of the VISTA ECD that are distinct from those of other Ig superfamily members, including two additional disulfide bonds, the insertion of an unstructured C-C’ loop, the striking enrichment of histidine residues within the ECD, and an extra H β-strand that forms an intramolecular clamping disulfide bond (79, 80). Mutagenesis studies have demonstrated that these structural features contribute to the surface orientation and suppressive function of VISTA (79, 80).

VISTA expression in mice is largely restricted within the hematopoietic compartment, with the highest expression on CD11b^+^ myeloid lineages such as monocytes, macrophages, granulocytes, and dendritic cells (73, 74). VISTA is also expressed in lymphocytes including NK cells, TCRγδ T cells, naïve CD4^+^ and CD8^+^ TCRαβ T cells, and Foxp3^+^ Tregs. A similar expression pattern of VISTA is seen in human peripheral blood monocytic cells. VISTA gene expression is positively regulated by the transcription factors P53, HIF1-α, and STAT3 (81–83). However, whether VISTA exerts any impact on the functions of HIF1-α and STAT3 remains unknown. VISTA expression is also regulated by TGF-β/Smad3 signaling in T cells and myeloid cells (84).

In human cancer tissues, VISTA expression was mostly enriched in tumor-infiltrating myeloid cells and T cells (75, 85). In addition to immune cells, VISTA expression was detected in mesothelioma (86), gastric cancer (87), and AML (83, 88, 89). VISTA expression has been associated with resistance to immunotherapy and poor patient survival in many cancers, including prostate cancer, lymphoma, bladder cancer, melanoma, breast cancer, and AML (88, 90–95),

### Molecular mechanisms of VISTA

VISTA impairs antitumor immunity through its ligand activity in myeloid cells and T cell-intrinsic activity. Although it has been speculated that VISTA also acts as an inhibitory receptor (96), the signaling mechanism is unclear and it remains possible that T cell-intrinsic activity may rely on *cis* interactions with other signaling partners. At the molecular level, several partners, such as PSGL-1, VSIG3, and galectin-9, have been identified to engage VISTA (97–99). While PSGL-1 was suggested as an inhibitory receptor for VISTA, VSIG3 was considered a ligand. Galectin-9 binds VISTA and forms a protein complex that promotes galectin-9-mediated apoptotic signaling. At the cellular level, VISTA regulates the development and function of macrophages, MDSCs, neutrophils, TCRγδ T cells, and CD4^+^/CD8^+^ conventional T cells (74, 75, 78, 100, 101). In macrophages, VISTA impairs TLR signaling by regulating the ubiquitination and stability of TRAF6 (102). Blocking VISTA synergizes with a TLR-agonistic vaccine by augmenting the activation of DCs and macrophages, increasing the production of stimulatory cytokines such as IL-12 and IL-27, and promoting the effector function of tumor-specific CTLs. VISTA also contributes to the suppressive function of MDSCs, although the exact molecular mechanisms remain undefined (82, 102).

### Combinatorial blockade of VISTA and PD-1

In preclinical models, genetic deletion of VISTA or treatment with anti-VISTA mAb delayed tumor regression by inducing DC maturation, reducing the abundance of adaptive Foxp3^+^ Tregs, reducing the abundance of MDSCs, and augmenting the effector function and abundance of CTLs (73, 76).

Studies led by Liu et al. first established the nonredundant and synergistic role of VISTA and PD-1 in mounting immune responses against self and tumor antigens (103). In both B16 melanoma and CT26 colon tumor models, combinatorial treatment with anti-VISTA and anti-PD-L1 mAbs resulted in tumor regression and long-term survival in comparison to monotherapies (103, 104). A separate VISTA-blocking mAb, SG7, suppressed the interaction between VISTA and VSIG3 or PSGL-1 and showed efficacy in combination with PD-1 blockade in the MC38 colon tumor model (105). Finally, a unique role of VISTA in promoting naive T-cell quiescence has been identified (106). Accordingly, a study in a CT26 tumor model showed that a triple blockade of VISTA/PD-1/CTLA-4 could improve the efficacy of PD-1/CTLA-4 dual blockade by promoting antigen-presentation in myeloid cells and reducing the quiescent state of CTLs (107).

Several clinically relevant VISTA-blocking agents have been developed and entered clinical trials. VSTB112 (Janssen Inc) was the first anti-VISTA mAb tested in the clinic (NCT02671955). CA-170 (Curis Inc) is an orally available small molecule that has dual targeting activities against PD-L1/L2 and VISTA. In preclinical models, CA-170 rescued T-cell function similarly to PD-1 antagonists and inhibited the growth of B16 melanoma, CT26, and MC38 murine tumor models (108, 109). CA-170 was tested in a phase I trial (NCT02812875) and a phase II trial (Clinical Trials Registry-India CTRI/2017/12/011026) (110). CA-170 showed an excellent safety profile and encouraging clinical activity in classic Hodgkin lymphoma and advanced NSCLC (109). HMBD-002 (Hummingbird Bioscience) is a human VISTA-specific mAb that binds to the C-C’ loop of VISTA and blocks its interaction with VSIG3 (111). Studies of murine and humanized mouse models showed the effects of HMBD-002 in reducing MDSCs and augmenting T-cell responses. The phase I trial of HMBD-002 is ongoing (NCT05082610). W0180 (Pierre Fabre Inc) is a human VISTA-specific mAb being tested in a phase I trial (NCT04564417). The NCT05082610 and NCT04564417 trials will both test VISTA inhibitors in combination with pembrolizumab. KVA12123 (Kineta Inc) is a third human VISTA-targeting mAb that has recently been granted FDA acceptance for testing in phase I/II trials.

## TIGIT

### TIGIT structure and expression

T-cell immunoreceptor with Ig and ITIM domains (TIGIT) is an ICR that contains an IgV-like ECD, a type I transmembrane domain, and a cytoplasmic domain with ITIM and ITT motifs (112). TIGIT is expressed on NK cells, CD4^+^/CD8^+^ conventional T cells, and Foxp3^+^ Tregs. In T cells, TIGIT expression is upregulated following TCR activation and is sustained with increased exhaustion (112).

In human cancers, TIGIT gene expression was found to be upregulated in tumors and correlated with poor prognosis for KIRC, KIRP, LGG, and UVM cancers (113). TIGIT protein expression is abundant in CD4^+^/CD8^+^ TILs and Tregs from a wide range of cancer types and is often correlated with poor clinical outcomes or resistance to ICI therapies (114). Coexpression of TIGIT and PD-1 on CD8+ TILs, which is associated with dysfunctional antitumor immune responses, has also been observed in cancers such as HCC, glioblastoma (GBM), acute myeloid leukemia, NSCLC, and melanoma (114).

### Molecular mechanisms of TIGIT

TIGIT binds to three ligands CD112, CD113, and PVR(CD155), out of which CD155 exhibits the highest affinity (115, 116). The TIGIT/CD155 interaction inhibits the functions of NK cells, T cells, and APCs. Phosphorylation of both the ITT and ITIM domains is required for the inhibitory signaling of TIGIT in NK cells and T cells, partly by recruiting the adaptors Grb2 and SHIP1, which in turn dampen the PI3K, MAPK, and NF-_K_B signaling pathways (117, 118). TIGIT also outcompetes CD226 for binding to CD155 and disrupts the costimulatory signal from CD226 in T cells (119). In addition to effector T cells, TIGIT is expressed in Foxp3^+^ Tregs and plays a role in promoting their differentiation, stability, and suppressive function (120–122).

In APCs such as DCs and macrophages, CD155 is phosphorylated upon engaging TIGIT and subsequently inhibits MAPK signaling, resulting in tolerogenic APCs that produce elevated levels of IL-10 but reduced levels of IL-12, and fail to properly stimulate cognate T cells (123). Another recent study demonstrated that leukemia-associated macrophages express TIGIT and that blocking TIGIT drives M1-like phenotypes and increases phagocytosis (124).

### Combinatorial blockade of TIGIT and PD-1

The efficacy of the dual blockade of TIGIT and PD-L1 has been demonstrated in murine breast and colon carcinoma models (112). The combination therapy rejuvenated tumor-specific CD8^+^ CTLs by augmenting their expansion, effector functions, and the development of memory responses (112). A recent study has shown that the PD-1 and TIGIT pathways converge to regulate CD226, as both receptors impair the phosphorylation of CD226 (125). Furthermore, when CD8^+^ TILs from human liver cancers were treated with TIGIT and PD-1-blocking mAbs, the coblockade of TIGIT and PD1 significantly improved the expansion, cytokine production, and cytotoxicity of CD8^+^ TILs compared with single PD-1 blockade (126). Similar results were seen in an adoptive T-cell transfer study to treat human lung cancer, where dual blockade of TIGIT/PD-1 or TIM-3/PD-1 resulted in greater tumor control than PD-1 monotherapy (127). Together, these studies provide a strong rationale for blocking both the PD-1 and TIGIT pathways to allow optimal CD226-dependent costimulatory signaling in CD8^+^ T cells.

Currently, there are approximately > 50 clinical trials in the US testing several TIGIT-targeted mAbs, either as monotherapy or in combination with PD-L1/PD-1 inhibitors (clinicaltrials.gov). Bispecific antibodies targeting both TIGIT and PD-1 are also being tested in these trials. In a phase II clinical trial sponsored by Roche (NCT03563716), anti-TIGIT mAb (Tiragolumab) was granted breakthrough therapy designation and was tested in combination with anti-PD-L1 (atezolizumab) in metastatic NSCLC (128). The combination treatment has improved the overall response rate, progression-free survival, and overall survival, over atezolizumab alone (128). Notably, the benefit of the combination treatment was mainly observed in patients with high PD-L1 expression (> 50%) (128, 129). Another TIGIT antibody Vibostolimab (MK-7684) was evaluated in a phase I trial (NCT02964013) with and without combination with pembrolizumab for advanced solid tumors, including NSCLC, and showed promising antitumor activity (130). Additional TIGIT inhibitors, such as BMS-986207 (NCT04570839) (131), ASP8374 (NCT03260322, NCT04826393) (132), Domvanalimab (AB154) (NCT04262856) (133), BGB-A1217 (NCT04047862) (134), and Etigilimab (OMP-313M32) (NCT04761198) (135) are under investigation as single agents and in combination with PD-1/PD-L1 inhibitors in solid tumors.

## TIM-3

### TIM-3 structure and expression

T-cell immunoglobulin and mucin domain-containing protein 3 (TIM-3), along with TIM1 and TIM4, belongs to the TIM family of immunoregulatory proteins. The ECD of TIM-3 contains an immunoglobulin variable domain that binds to several ligands: galectin 9, phosphatidylserine, CEACAM1, and HMGB1 (136). Following the ECD is a mucin domain, a transmembrane domain, and a cytoplasmic domain that does not contain canonical inhibitory signaling motifs such as ITIM or ITSM motifs.

TIM-3 is expressed on subsets of activated CD4^+^ and CD8^+^ conventional T cells, Foxp3+ Tregs, NK cells, myeloid cells, and mast cells (136). TIM-3 can be cleaved into a soluble form by ADAM10 and ADAM17 (137). TIM-3 expression in T cells is coregulated with other ICRs including PD-1, TIGIT, and LAG-3 (138). Cytokines such as IL-12, IL-27, and IFN-β can upregulate TIM-3 expression (139, 140). In human cancers, TIM-3 is highly expressed in terminally exhausted CD8^+^ CTLs, Foxp3^+^ Tregs, tumor-associated macrophages, and MDSCs. TIM-3 expression levels have been shown to correlate with resistance to immunotherapies and poor prognosis in many cancer types such as melanoma, HCC, prostate cancer, RCC, colon cancer, bladder cancer, cervical cancer, gastric cancer, and esophageal squamous cell carcinoma (122, 141–149).

### Molecular mechanisms of TIM-3

In conventional T cells, TIM-3 is recruited to the immune synapse upon TCR activation (150). Y256 and Y263, two of the five tyrosines on the cytoplasmic tail of TIM3, bind BAT3, a protein involved in the TCR signaling pathway (151). Bound BAT3 recruits LCK, a major upstream player in the TCR signaling pathway (152). However, engagement with galectin 9 results in the phosphorylation of Y256 and Y263 by interleukin-2-inducible T-cell Kinase (ITK), which releases BAT3 and impairs TCR signaling (153, 154). Another ligand, CEACAM1, binds TIM-3 *in cis* to promote the stability of TIM-3, while the *trans* interaction induces similar signaling outcomes as galectin-9 (154). The Galectin 9/TIM-3 axis induces apoptosis of effector Th1 cells and CD8^+^ CTLs (152, 155, 156). In Foxp3^+^ Tregs, TIM-3 signaling drives an effector-like phenotype and enhances suppressive function (157).

TIM-3 is also expressed in DCs, where its ligation induces the activation of Bruton’s tyrosine kinase and c-Src, which inhibit NF-kB activation and subsequently reduce DC activation (158). In macrophages, TIM-3 has been reported to promote M2-like polarization by inducing SOCS1 (159). In monocytes and DCs, TIM-3 inhibits the cellular responses to TLR signaling and reduces the production of proinflammatory mediators (160). In a breast cancer model, blocking TIM-3 augmented the production of a key chemokine CXCL9 from CD103^+^ DCs, thereby improving the antitumor immune responses when combined with chemotherapy (161).

### Combinatorial targeting of TIM-3 and PD-1

In preclinical models, dual blockade of TIM-3 and PD-1 restored the function of both CD4^+^ and CD8^+^ T cells and led to complete tumor regression whereas either monotherapy was not effective (162, 163). A recent study has shown that PD-1 binds galectin-9 and that PD-1/TIM-3/galectin-9 may crosslink and form a lattice. As such, PD-1 functions to attenuate galectin-9/TIM-3-induced apoptosis (164). It should be noted that VISTA also binds to galectin-9 and augments the inhibitory effects of TIM-3 (99). Thus, these findings may provide a rationale for future studies to test the combined blockade of PD-1, TIM-3, and VISTA, to improve the persistence and functions of tumor-reactive PD-1^+^ TIM-3^+^ CTLs.

In human cancers, TIM-3 and PD1 are often coexpressed on CD8^+^ T cells and mark the most dysfunctional T cell subsets. An earlier study of advanced melanoma showed that NY-ESO-1-specific PD-1^+^CD8^+^ TILs upregulate TIM-3 expression, which is correlated with dysfunctional phenotypes (165). Blocking TIM-3 augmented cytokine production and proliferation of T cells, while combined blockade of both TIM-3 and PD-1 showed synergistic effects. Similar findings were reported in colorectal cancer, where TIM-3^+^PD-1^+^CD8^+^ TILs represented the predominant fraction of TILs and targeting both TIM-3 and PD-1 enhanced cell expansion, cytokine production, and cytotoxic activity (166). Recent studies of diffuse large B-cell lymphoma found that TIM-3^+^PD1^+^ TILs exhibited a transcriptomic signature of T-cell exhaustion, reduced proliferation, and impaired cytokine production, but these dysfunctions were restored by the blockade of PD1 or TIM-3 (167, 168). Although there have not been any FDA-approved therapeutics targeting TIM-3, the pipelines for novel TIM-3 inhibitors are expanding: several TIM-3-specific antibodies (i.e., cobolimab, MBG453, Sym-023, BMS-986258, AZD7789, INCAGN02390, etc.) or TIM-3/PD-1 bispecific antibodies are being tested in clinical trials (169). A phase I/II trial (NCT02608268) evaluated MGB453 (anti-TIM3) in combination with PDR001 (anti-PD-1) in advanced solid cancers such as melanoma and NSCLC and showed excellent safety profile and preliminary antitumor activity (170). Similar encouraging results were shown by trials (NCT02817633 and NCT03680508) that evaluated TSR-022 (anti-TIM3) in combination with PD-1 inhibitors (171, 172). In addition, a phase Ia/b trial evaluated the safety, pharmacokinetics, and efficacy of LY3321367 (anti-TIM3) plus LY3300054 (Anti-PD-L1) and showed modest antitumor activity (173).

## LAG-3

### LAG-3 structure and expression

Lymphocyte activation gene 3 (LAG-3, CD223) is an Ig superfamily ICR and is homologous to CD4 (174, 175). The ECD of LAG-3 contains four IgV or IgC-like domains that are involved in ligand binding. The cytoplasmic domain of LAG-3 contains a serine phosphorylation site, the conserved KIEELE motif, and the glutamate-proline dipeptide repeat motif that is involved in its inhibitory signaling (176).

LAG-3 is expressed in many immune cell types including activated conventional CD4^+^/CD8^+^ T cells, Foxp3^+^ Tregs, TCRγδ T cells, NK cells, dendritic cells, and B cells (175). In T cells, LAG-3 expression is induced upon TCR stimulation or by cytokines such as IL-12, IL-2, IL-15, IL-7, IL-6, and IL-8 (177–179). LAG3 expression is promoted by transcription factors such as TOX, NFAT, and NR4A, while suppressed by T-bet (180–186). Studies of human cancers have shown that LAG-3 expression is abundant in TILs and associated with T cell dysfunction or insensitivity to PD-1 blockade. These include breast cancer (187), kidney renal clear cell carcinoma (188), melanoma (189), NSCLC (190, 191), HCC (192, 193), and B-cell lymphoma (194). LAG-3 expression in peripheral blood lymphocytes is also associated with resistance to ICI therapies in patients with melanoma and urothelial carcinoma (195). Furthermore, the clinical resistance to PD1 blockade may be correlated with reduced shedding of LAG-3 in CD4^+^ conventional T cells due to reduced expression of the protease ADAM10 (196).

### Molecular mechanisms of LAG-3

LAG-3 is recognized by multiple ligands including MHCII (197–199), fibrinogen-like protein 1 (FGL-1) (200), galectin-3 (201), and liver sinusoidal endothelial cell lectin (LSECtin) (202). In conventional T cells, LAG-3 signaling suppresses T cell activation, proliferation, cytokine secretion, and cytotoxic functions (203). LAG-3 interacts *in cis* with the TCR/CD3 complex and inhibits TCR signaling by promoting local acidification and Lck dissociation (204). LAG-3 and PD1 interact *in cis* and cluster with pLck at the immunological synapse and recruit SHP1/2, thereby exerting inhibitory effects on T-cell signaling (205). LAG-3 also promotes the activation and suppressive function of Foxp3^+^ Tregs (206). Soluble LAG-3 acts as an MHCII agonist and induces tyrosine phosphorylation and activation of the AKT and ERK1/2 signaling pathways, thereby inducing DC maturation and improving antitumor T-cell responses (207, 208).

### Combinatorial targeting of LAG-3 and PD-1

Preclinical studies have established that LAG-3 cooperates with PD-1 in controlling antitumor immunity (175, 209). The striking synergy between LAG-3 and PD-1 has been demonstrated in murine melanoma, colon cancer, and ovarian tumor models, where the dual blockade against LAG-3 and PD-1 effectively controlled tumor progression that was resistant to respective monotherapies (205, 210). A study in the MC38 colon cancer model has shown that PD-L1 blockade elevated the expression of both costimulatory receptors (ICOS) and coinhibitory receptors (LAG3 and PD-1) in TILs, thereby providing a new mechanistic rationale for coblocking LAG3 (211).

In human ovarian cancer, NY-ESO-1-specific CD8^+^ TILs demonstrated impaired effector function and enriched coexpression of the inhibitory molecules LAG-3 and PD-1. Dual blockade of LAG-3 and PD-1 during T-cell priming efficiently augmented proliferation and cytokine production by NY-ESO-1-specific CD8^+^ T cells (212).

These preclinical and clinical studies have provided the backbone for combinational treatment strategies. Currently, numerous clinical trials are exploring the therapeutic benefits of simultaneously targeting LAG-3 and PD-1 (209, 213). LAG-3 targeted agents include soluble LAG-3, LAG-3-specific mAbs, or bispecific antibodies recognizing both LAG-3 and PD-1. Relatlimab (anti-LAG-3) in combination with nivolumab received FDA approval in March 2022 for treating unresectable or metastatic melanoma (214). Favezelimab (MK-4280) in combination with pembrolizumab was tested in a phase III trial (NCT02720068) for colorectal cancer and showed promising antitumor activity in PD-L1-positive tumors (213, 215). Ieramilimab (LAG525) was tested in a phase I/II study (NCT02460224) in combination with spartalizumab (PDR001, anti-PD-1) in advanced/metastatic solid tumors such as melanoma and TNBCs, demonstrating a good toxicity profile but moderate antitumor activity (216). Fianlimab (REGN3767, anti-LAG3) is being tested in combination with cemiplimab (anti-PD-1) in a phase I dose-escalation study (NCT03005782) in advanced melanoma patients and showed clinical activities (217). Eftilagimod alpha, a soluble LAG-3 fusion protein, is being tested along with pembrolizumab for treating recurrent or metastatic head and neck squamous cell carcinoma (NCT03625323) (208). An ongoing phase I/II study (NCT04370704) is testing retifanlimab (INCMGA00012, Anti–PD-1), INCAGN02385 (Anti–LAG-3), and INCAGN02390 (Anti–TIM-3) triple combination therapy in patients with advanced solid tumors (218). Multiple trials tested BI-754111 (anti-LAG3) combined with BI-754091 (anti-PD-1) in patients with advanced solid tumors but no significant antitumor activity was reported (219). Lastly, bispecific antibodies targeting PD-1/LAG3, including tebotelimab (MGD013, NCT04212221) and RO7247669 (NCT04140500) are under early-stage clinical investigations (220).

## Conclusions

Since the first FDA approval of ICIs in 2011, significant progress has been made toward optimizing existing ICI therapies. Taking the lessons from existing ICIs that target PD-1, PD-L1, and CTLA-4, current efforts in the field focus on identifying and targeting nonredundant ICRs that may potentially synergize with existing therapies. VISTA, TIGIT, TIM-3, and LAG-3 represent such candidates in the pipeline. Recent advances in understanding the converging role of ICRs in driving the dysfunction of both APCs and T cells (**Figures 1**, **2**) have set the conceptual foundation for developing combinatorial therapies targeting these ICRs. Based on the frequent coexpression of ICRs in tumor tissues and the distinct yet convergent mechanisms of action (**Table 1**), it is expected that combined blockade of these emerging ICRs with PD-L1/PD-1 will result in additive or synergistic outcomes. Indeed, many novel ICI combination therapies are being investigated in early-stage trials (**Table 2**). To advance this concept into clinical applications, the field still faces some challenges, such as defining the molecular pathways and hierarchy of emerging ICRs, identifying the optimal ICR combinations for distinct cancer types and discrete biomarkers, and developing better preclinical models that present the full extent of immune-related toxicities as seen in human patients. In conclusion, we emphasize that antitumor immunity is controlled by multiple nonredundant ICRs that together maintain immune dysfunction. Recent preclinical and early clinical data strongly support the rational design of novel ICI combinations to achieve synergistic therapeutic efficacies with manageable toxicities.

**Table 1:** Blocking individual ICRs augments antitumor immune responses by convergent cellular and molecular mechanisms.

Effect in immune cell	PD-1 blockade	CTLA-4 blockade	VISTA blockade	TIGIT blockade	TIM3 blockade	LAG3 blockade
**Conventional T cells**	Augment CD28- mediated costimulation; enhance the proliferation and effector function of CTLs; Expand progenitor-like exhausted CTLs.	Expand ICOS+Th1-like CD4+ effector T cells; expand terminally differentiated effector CD8+ CTLs; expand CTL TCR repertoire; enhance CTL effector function; Improve T cell stop signal and interaction with DCs; combined blockade with anti-PD1 obtain synergistic effects	Enhance CTL cell proliferation, cytokine production and cytotoxic function; reduced CTL quiescence; combined blockade with anti-PD1 obtain synergistic effects.	Enhance CTL cell proliferation, cytokine production and cytotoxic function; combined blockade with anti-PD1 obtain synergistic effects.	Enhance CTL cell proliferation, cytokine production and cytotoxic function; improve T cell survival; combined blockade with anti-PD1 obtain synergistic effects	Enhance CTL cell proliferation, cytokine production and cytotoxic function; combined blockade with anti-PD1 obtain synergistic effects
**FOXP3+ Tregs**	Induces hyper-expansion of Tregs and contribute to hyper-progressive diseases.	Reduce intratumoral Tregs.	Reduce the differentiation of adaptive Tregs and their suppressive functions	Reduce Treg stability and suppressive function		Reduce the suppressive activity of Tregs
**Antigen presenting cells (APCs)**	Augment macrophage phagocytosis and M1 polarization.	Increase surface expression of B7 on APCs; reduce IDO expression	Promotes antigen presentation in DCs and macrophages; promote TLR-mediated activation and cytokine production of DCs and macrophages	Promotes M1 polarization of macrophages and DC activation; increase the production of chemokine Cxcl9 and cytokines	Promotes M1 polarization of macrophages; TLR signaling; DC activation	Soluble LAG-3 acts as a MHCII agonist and induces DC activation
**Myeloid derived suppressor cells (MDSCs)**	Reduce the expansion of tumor driven GMP and MDSCs; augment ERK1/2 and mTORC1 signaling; metabolic reprogramming in myeloid progenitors.		Reduce the abundance and suppressive function of MDSCs.			

This table summarizes the multitudinous effects of blocking each ICR, including PD-1, CTLA-4, VISTA, TIGIT, TIM-3, and LAG-3, in regulating antitumor immune responses. The relevant effector cell types include effector T cells, Foxp3^+^ Tregs, APCs, and MDSCs.

**Table 2:** Clinical trials testing combined targeting of ICRs.

ICI combinations	Agents	Company	Clinical trials	Cancer types
**CTLA4 + PD-1/PD-L1**	Ipilimumab+ Nivolumab	Bristol-Myers Squibb	FDA approval	HCC, pleural mesothelioma, metastatic melanoma, colon cancer, urothelial cancer, metastatic NSCLC, RCC
	Ipilimumab+ Nivolumab	Bristol-Myers Squibb	NCT04080804 NCT04606316	HNSCC Glioblastoma Results: recruiting
	Ipilimumab+ Pembrolizumab	Merck Sharp & Dohme	NCT02089685 NCT03302234 NCT03873818	Metastatic melanoma, RCC Results: showed tolerability and antitumor activity NSCLC Results: combination therapy failed to improve efficacy over monotherapy. Metastatic melanoma (recruiting)
**VISTA + PD-1/PD-L1**	CA170 (dual activity)	Curis	NCT02812875 CTRI/2017/12/011026	Hodgkin lymphoma, NSCLC No results
	HMBD-002 + Pembrolizumab	Hummingbird	NCT05082610	Advanced solid tumors, TNBC, NSCLC No results
	W0180 + Pembrolizumab	Pierre Fabre	NCT04564417	Locally advanced or metastatic solid tumors, No results
	KVA12123 + pembrolizumab	Kineta	NCT05708950	Advanced solid tumors, Recruiting
**TIGIT + PD-1/PD-L1**	Tiragolumab + Atezolizumab	Roche	NCT03563716	Metastatic NSCLC Results: show improved ORR and PFS
	Vibostolimab (MK-7684) + Pembrolizumab	Merck Sharp & Dohme	NCT02964013 NCT04725188 NCT04738487 NCT05665595 NCT02625961 NCT05298423 NCT05845814	Advanced solid tumors, including NSCLC, melanoma, bladder cancer, urothelial carcinoma Results: recruiting
	BMS-986207 + Nivolumab+ Ipilimumab	Bristol-Myers Squibb	NCT05005273	NSCLC Results: terminated
	BMS-986207 + Nivolumab+ COM701 (anti- PVRIG)	Bristol-Myers Squibb	NCT04570839	Advance solid tumors No results
	ASP8374 + Pembrolizumab	Astellas	NCT03260322 NCT04826393	Advance solid tumors Recurrent glioma No results
	Domvanalimab (AB154) + Zimberelimab (AB122, anti-PD-1)	Arcus Bioscience	NCT04262856	Metastatic NSCLC Results: improved ORR and PFS in combination therapy.
	BGB-A1217 + Tislelizumab (anti-PD-1)	Beigene	NCT04047862	metastatic squamous NSCLC Results: recruiting
	Etigilimab + Nivolumab	Mereo BioPharma	NCT04761198	Advanced solid tumors, cervical cancer, uveal melanoma, ovarian cancer, NSCLC. Results: showed early efficacy
**TIM3 + PD-1/PD-L1**	Cobolimab (TSR-022) + Nivolumab or TSR-042 (anti-PD-1)	Tesaro	NCT02817633 NCT03680508	Advanced solid tumors such as NSCLC, melanoma, HCC, Results: showed clinical efficacy
	Sabatolimab (MBG453) + Spartalizumab (PDR001, anti-PD-1)	Novartis	NCT02608268	Advanced solid cancers such as melanoma and NSCLC Results: preliminary antitumor activity
	Sym023 + Sym-021 (anti-PD-1)	Symphogen	NCT03311412	Advanced solid tumors, lymphomas, No results.
	LY3321367 + LY3300054 (Anti-PD-L1)	Eli Lilly	NCT03099109	Advanced solid tumors, Results: modest antitumor activity.
	BMS986258 + Nivolumab	Bristol-Myers Squibb	NCT03446040	Advanced solid tumors, Recruiting
**LAG3 + PD-1/PD-L1**	Relatlimab + Nivolumab	Bristol-Myers Squibb	FDA approval	Unresectable or metastatic melanoma
	Favezelimab (MK-4280) + Pembrolizumab	Merck Sharp & Dohme	NCT02720068 NCT03598608 NCT05064059	Colorectal cancer, Lymphomas, Recruiting
	Ieramilimab + Spartalizumab (PDR001, anti-PD-1)	Novartis	NCT02460224	Advanced solid tumors, melanoma, TNBCs, mesothelioma, Results: modest antitumor activity
	Fianlimab + Cemiplimab (anti-PD-1)	Regeneron	NCT03005782	Advanced melanoma, Results: preliminary antitumor activity, ongoing biomarker analysis
	Eftilagimod alpha + Pembrolizumab	Immutep	NCT03625323	Metastatic NSCLC and HNSCC, Results: showed antitumor activity
	Encelimab (TSR-033) + Dostarlimab (TSR-042, anti-PD-1)	Tesaro	NCT03250832	Advanced solid tumors, No results.
	BI-754111 + BI-754091 (anti-PD-1)	Boehringer Ingelheim	NCT03156114 NCT03433898 NCT03697304 NCT03780725	Advanced solid tumors, NSCLC, Results: manageable safety profile but no improved antitumor activity
	Sym-022 + Sym-021 (anti-PD-1)	Symphogen	NCT03311412 NCT03489369 NCT03489343	Advanced solid tumors, lymphomas, Results: preliminary antitumor activity
**LAG3 + TIM3 +PD-1**	INCAGN02385 (anti-LAG3) + INCAGN2390 (anti-TIM3) + Retifanlimab (INCMGA00012, Anti–PD-1)	Incyte	NCT04370704	Advanced solid tumors Results: recruiting

## Author contributions

DR: Writing – original draft, Writing – review & editing. CG: Writing – original draft, Writing – review & editing. SP: Writing – original draft, Writing – review & editing. LW: Writing – original draft, Writing – review & editing, Conceptualization, Funding acquisition, Project administration, Supervision, Validation.
